# Nociceptive Transmission to Rat Primary Somatosensory Cortex – Comparison of Sedative and Analgesic Effects

**DOI:** 10.1371/journal.pone.0053966

**Published:** 2013-01-08

**Authors:** Marcus Granmo, Tanja Jensen, Jens Schouenborg

**Affiliations:** Neuronano Research Center, Department of Experimental Medical Sciences, Lund University, Lund, Sweden; University of Pittsburgh, United States of America

## Abstract

CO_2_-laser C-fibre evoked cortical potentials (LCEPs) is a potentially useful animal model for studies of pain mechanisms. A potential confounding factor when assessing analgesic effects of systemically administered drugs using LCEP is sedation. This study aims to clarify: 1) the relation between level of anaesthesia and magnitude of LCEP, 2) the effects of a sedative and an analgesic on LCEP and dominant EEG frequency 3) the effects of a sedative and analgesic on LCEP when dominant EEG frequency is kept stable. LCEP and EEG were recorded in isoflurane/nitrous-oxide anaesthetized rats. Increasing isoflurane level gradually reduced LCEPs and lowered dominant EEG frequencies. Systemic midazolam (10 **μ**mol/kg) profoundly reduced LCEP (19% of control) and lowered dominant EEG frequency. Similarly, morphine 1 and 3 mg/kg reduced LCEP (39%, 12% of control, respectively) and decreased EEG frequency. When keeping the dominant EEG frequency stable, midazolam caused no significant change of LCEP. Under these premises, morphine at 3 mg/kg, but not 1 mg/kg, caused a significant LCEP reduction (26% of control). In conclusion, the present data indicate that the sedative effects should be accounted for when assessing the analgesic effects of drug. Furthermore, it is suggested that LCEP, given that changes in EEG induced by sedation are compensated for, can provide information about the analgesic properties of systemically administrated drugs.

## Introduction

To develop new analgesics, appropriate animal models of pain are crucial. The current models are based primarily on measuring changes in motor responses [1]–[9]. Because the nociceptive input to motor systems and to sensory systems are channelled through at least partly different central pathways, with different physiological and pharmacological properties [10]–[11], the validity of motor responses in predicting sensory aspects of pain and analgesia is not always clear [11]–[13]. To develop new and effective analgesics, it is therefore crucial to develop supplementary animal models that provide assessments of the activity in the brain regions involved in the sensory aspects of pain.

Monitoring cortical potentials evoked by electrical or cutaneous CO_2_ laser stimulation in animals has shown that nociceptive C fibres provide powerful input to SI cortex [10], [12], [14]–[17]. This is mediated by multiple parallel spinal pathways in the rat [12], [16]. Notably, CO_2_ laser evoked C fibre potentials (LCEPs) are reduced following Morphine-induced spinal analgesia [18] and increased in an NMDA-dependent way after spinal wind-up [19]. Moreover, we recently found that LCEP can provide information on mechanisms related to primary and secondary UVB induced hyperalgesia [14]. Thus, rat LCEPs can be used to monitor pain related ascending transmission under various conditions and may provide a useful animal model for the assessment of potentially analgesic drugs. Since an analgesic drug also may possess sedative effects a key issue is whether one can differentiate between these two effects. To our knowledge, the sedative effects on nociceptive C fibre mediated input to the cortex are not known other than that LCEP are abolished by deep anaesthesia. The aims of the present study were 1) to clarify the relation between the level of anaesthesia and magnitude of LCEP, 2) to analyse the effects of a sedative (Midazolam) and an analgesic (Morphine) compound on the LCEP and the dominant frequency of EEG [20]–[23] and 3) to examine the effects of a sedative and an analgesic compound on LCEP in a situation where the dominant EEG frequency is kept stable.

## Methods

### Animals used

38 male Sprague-Dawley rats weighing 200–260 g were used. All animals received food and water *ad libitum* and were kept in a 12-h day-night cycle at a constant environmental temperature of 21°C (humidity 65%). Approval for the experiments was obtained in advance from the Lund/Malmoe local ethical committee on animal experiments, regulated by the code of regulations of the Swedish Board of Agriculture. These regulations, including directives from the European Union, follow the law on animal welfare legislated by the Swedish parliament. The County Administrative Board governs the implementation of the rules. Further, the experiments were in accordance with the policies and guidelines reported previously [24]–[25].

### Surgery and preparation for electrophysiology

The animals were anesthetized with Isoflurane (1.8–2.0% during surgery) in a mixture of 40% oxygen and 60% nitrous oxide. The trachea was cannulated and the end-expiratory pCO_2_ (3.0–4.5%) was continuously monitored. An infusion of 30–50 μl/min of 5% glucose in Ringer's acetate was given in the right jugular vein. Mean arterial blood pressure (80–150 mmHg) was monitored continuously in the right brachial artery. The rectal temperature was kept between 36.5–38.5°C using a feedback regulated heating system.

**Figure 1:**
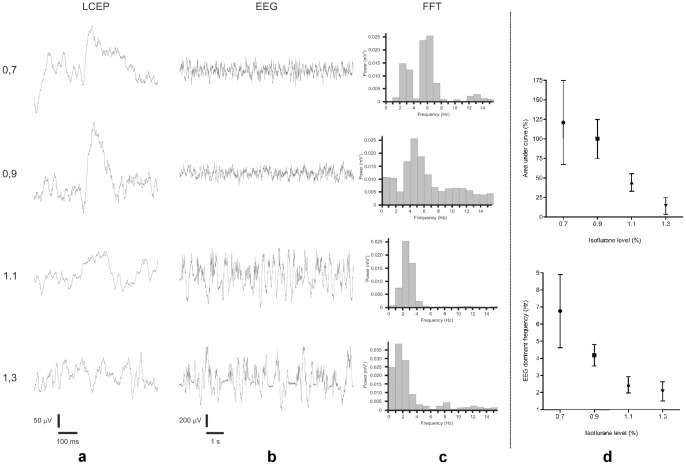
The effect of different Isoflurane concentrations (%) on LCEP (a) and EEG (b). Frequency distribution (FFT = fast Fourier transformation) of sampled EEG (c). (a–c) show averaged LCEP, raw data EEG sweeps and FFT from a single animal. (d) Normalized grand mean and standard deviation of averaged LCEP area under curve (AUC) and EEG dominant frequency recordings from five rats. 100% corresponds to the mean LCEP AUC at 0.9% isoflurane.

The spinous process of the 10^th^ thoracal vertebra was clamped and the chest lifted to facilitate ventilation. The head of the rat was fixed by ear bars and a nose ring. Pancuronium bromide (*Pavulon*, 0.5 mg) was given and the animal was artificially ventilated. Cerebrospinal fluid was drained between the base of the skull and the first cervical vertebra, to reduce the risk of cortical oedema. A craniotomy exposing the right parietal cortex was made. The *dura mater* of the parietal cortex was cut and covered with paraffin oil. Local infiltration of 2.0 mg/ml lignocaine (*Xylocaine*) with 1.2 μg/ml adrenaline was made during all surgery to reduce nociceptive input during surgery and to minimize possible postoperative excitability changes [26]. After completed surgery, the Isoflurane level was lowered to 0.8–0.9% in the same gas mixture as before. This anaesthetic level was characterized by an EEG dominated by 3–8 Hz theta waves (mean level, see fig. 1), with no signs of desynchronization during noxious stimulation. The blood pressure was stable also during noxious stimulation. Experiments were terminated after any signs of deterioration, i.e. precipitous drops in blood pressure or expiratory pCO_2_ levels.

### Stimulation and recordings; protocol and drug administration

**Figure 2:**
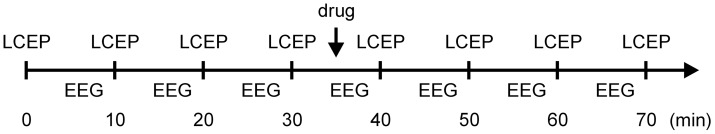
An overview of the stimulation protocol. LCEP stimulation was performed every 10 minutes. The first LCEP (t = 0) and the first LCEP after drug were not included in the data analysis (see methods). “EEG” mark the time period during which 2 separate EEG recordings were made.

Recordings of LCEPs and EEG were made from the contralateral cortical surface hindpaw representation area with fine silver ball-tipped electrodes (∼0.3 mm diameter). See the Data analysis section for filtering parameters. Tactile input was used to locate the cortical representation of the glabrous skin of the digits, arch and heel of the left hind paw [12], [14], [19]. A hand-held mechanical stimulator with a blunt metal probe (0.8 mm diameter) attached to a coil, was used for tactile stimulation. The probe was displaced by a current pulse generated by a Grass stimulator. The stimulation was adjusted to cause a light touch of the skin, without any visible joint movement. Radiant heat pulses emitted by a CO_2_-laser (Irradia, Sweden; wavelength 10.6 μm, output power 15 W, beam diameter 3.0 mm, pulse duration 20 to 32 ms) were used to elicit LCEP. These stimulation energies have previously been shown to reliably evoke late cortical field potentials (onset latency exceeding 180 ms) in rat SI through the activation of cutaneous nociceptive C fibres [14], [19]. No visible damage to the skin was observed using this stimulation. The pulse duration was adjusted to the local paw temperature (27–34°C) [27]. This corresponds to approximately 2–3 times the threshold for evoking LCEP. CO_2_ laser stimulation, consisting of trains of 16 pulses at a frequency of 1.0 Hz, of the glabrous skin of the hind paw was made to obtain averaged LCEPs. The stimulation sites were randomized in order to avoid repeated stimulation of the same sites (to avoid desensitization of C-nociceptors). In the beginning of each experiment, a baseline was obtained from at least 4 averaged LCEPs. The time interval between averages was set to 10 minutes. The first LCEP recording was not used in the analysis, as a stable baseline was obtained after the first train. EEG was sampled at regular intervals (for 45 s approximately every 5 minutes), always with at least two minutes pause after noxious stimulation. See figure 2 for an overview of the stimulation protocol.

After control recordings, Midazolam 10 μmol/kg or Morphine 1 or 3 mg/kg was administered through the right jugular vein. The drug doses used were within the range of effective doses found previously in various models of nociceptive transmission [28]–[30]. After drug, averaged LCEPs were collected as above. Due to pharmacodynamics the first averaged LCEP obtained 5 minutes after drug was not used. Instead, 3 separate averaged LCEPs starting at fifteen minutes after drug application was used for analysis. In some experiments the level of Isoflurane was lowered to 0.6–0.7% from 0.8–0.9% (the level of oxygen/nitrous oxide was kept constant throughout the experiments) after drug administration to reverse the dominant EEG frequency to control level (see data analysis section).

### Data analysis

The signals (10 kHz sampling frequency) were amplified and filtered using Digitimer Neurolog system (Digitimer LTD, England; 1–700 Hz sampling window) and collected using CED 1401 A/D converter and CED Signal software (Cambridge electronic design, Cambridge UK). The epoch length was 45 s for EEG recordings and 0.7 s with a pre-stimulus interval of 10 ms for evoked potentials. CO_2_ laser Aδ evoked potentials (onset latency 20–45 ms from start of stimulation), occurred irregularly and were therefore not analysed in detail. In case of C fibre input, due to their slow conduction velocity the impulses arrive to the spinal cord during a relatively long time period. Therefore, to obtain a representative measure of the magnitude of the activity evoked by nociceptive C fibres following a laser stimulus, the area under the curve (AUC) was calculated using in-house scripts created in Scilab-4.1.1 (INRIA, France). The AUC was defined as the sum of amplitudes between the baseline level and LCEP, with a maximum duration of 300 ms from the onset latency of LCEP (as found suitable from previous studies with the same setup [14], [19]. Baseline was set to the amplitude at the onset latency of each averaged LCEP. Animals were excluded from analysis if they did not show clear LCEPs or in the case where EEG frequency reversal was performed inadequately (4 rats). Off-line Fourier analysis (bin size 1024) was used to analyse the dominating EEG frequency, defined as the bin with the highest frequency power. As the data was found to follow a normal distribution (D'Agostino-Pearson omnibus test) we used the paired Student's t-test (two-tailed, 95% confidence interval) for statistical analysis, P<0.05 being considered as a statistically significant difference. Statistical analysis was also performed to ascertain that the baseline dominant frequency of EEG was similar between the different experimental groups (unpaired student's t-test with Welch's correction, see table 1). In order to facilitate comparison of data between animal groups, all data within each group are normalized to its respective baseline measurement mean (see table 2 for data before normalization).

**Table 1:** Statistical analysis between experimental groups of LCEP AUC baselines before drug.

Comparison	P-value
Midazolam no comp *vs* with comp	0.73
Morphine 1 mg/kg no comp *vs* with comp	0.21
Morphine 1 mg/kg no comp *vs* 3 mg/kg no comp	0.51
Morphine 1 mg/kg no comp *vs* 3 mg/kg with comp	0.10
Morphine 1 mg/kg with comp *vs* 3 mg/kg no comp	0.41
Morphine 1 mg/kg with comp *vs* 3 mg/kg with comp	0.32
Morphine 3 mg/kg no comp *vs* 3 mg/kg with comp	0.18

Results from unpaired student's t-test with Welch's correction for unequal variances is shown. No significant differences between the groups were found.

**Table 2:** LCEP area under curve (AUC) mean values before normalization (see methods) and corresponding standard deviation (SD) from the different set of experiments.

Isoflurane	0,7%	0,9%	1,1%	1,3%
AUC	90,3	74,6	32,9	10,6
SD	40,1	18,5	8,4	8,0
**Midazolam**	**Before**	**After**	**Before comp**	**After comp**
AUC	66,9	20,6	68,7	58,8
SD	17,6	3,8	15,8	10,5
**Morphine 1 mg/kg**				
AUC	94,1	37,4	58,0	43,3
SD	14,3	15,0	24,5	16,7
**Morphine 3 mg/kg**				
AUC	84,1	0,3	65,2	20,7
SD	29,2	16,8	23,3	11,9

Before comp/after comp = experiments with EEG dominant frequency compensation.

## Results

All analysis data below were obtained from averaged LCEPs. Consistent with previous studies [14], [19], these potentials consisted of a late surface positive potential with relatively constant onset latency for each stimulation area in each rat (stimulation of digits, mean onset latency 250 ms, SEM 10 ms; arch, mean onset latency 235 ms, SEM 17 ms; heel, mean onset latency 220 ms, SEM 13 ms).

### Effect of Isoflurane on LCEP and dominant frequency of EEG

Initially we tested the effect of different concentrations (0.7, 0.9, 1.1 and 1.3%) of Isoflurane on EEG and averaged LCEP (Figure 1) in five rats. At the level of anaesthesia previously used (0.8–0.9% Isoflurane), the EEG was dominated by theta waves (3–8 Hz) and clear LCEPs could be elicited. As can be seen, averaged LCEP and EEG rhythm were gradually reduced when the concentration of Isoflurane was increased up to 1.3%. At this point averaged LCEP was nearly abolished and the mean dominating frequency of EEG was 2.0 Hz.

### The effect of systemic administration of Midazolam 10 μmol/kg on LCEP and EEG

**Figure 3:**
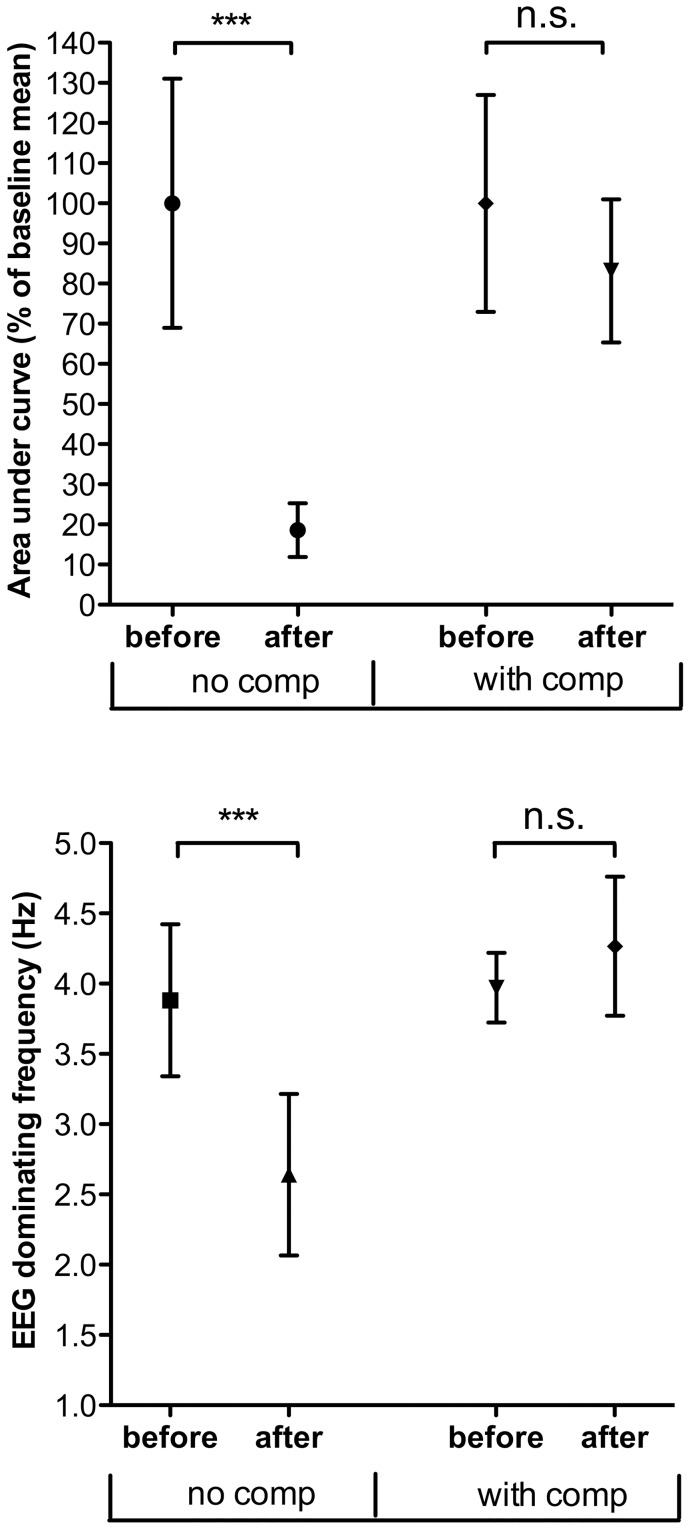
The effect of Midazolam on LCEP and EEG. Normalized grand mean and standard deviation of averaged LCEP area under curve and EEG dominant frequency recordings is shown. “With comp” represents experiments with adjustment of volatile anaesthesia to keep a stable dominant EEG frequency after drug. “before” represents baseline measurements before drug and “after” represents LCEPs elicited after drug administration. The results from student's t-test are also shown. See methods section for details.

It is well known that sedative compounds can inhibit nociceptive pathways in an unspecific way. In a set of experiments (nine animals) LCEPs were sampled before and after systemic administration of Midazolam 10 μmol/kg. Midazolam resulted in a markedly reduced averaged LCEP AUC (18,6% of control; Student's t-test P<0.0001, degrees of freedom df = 8) and a shift of the dominant EEG frequency towards lower frequencies (Figure 3, dominating frequency 3.9 versus 2.6 Hz; Student's t-test P = 0,0006, df = 8), resembling the effect of increasing the Isoflurane concentration from 0.9 to 1.1% (Figure 1).

In seven rats, the dominant frequency of EEG after drug administration was kept stable at control level (Figure 3) by lowering the percentage of the volatile anaesthetic used (Isoflurane), from 0.8–0.9 to 0.6–0.7%. In this situation, there was no significant change in averaged LCEP after Midazolam treatment as compared to control (AUC 83% of control, Student's t-test P = 0.1571, df = 6) thus suggesting that the apparent analgesic effect of Midazolam is mainly sedative (Figure 1).

### The effect of systemic administration of Morphine on LCEP and EEG

**Figure 4:**
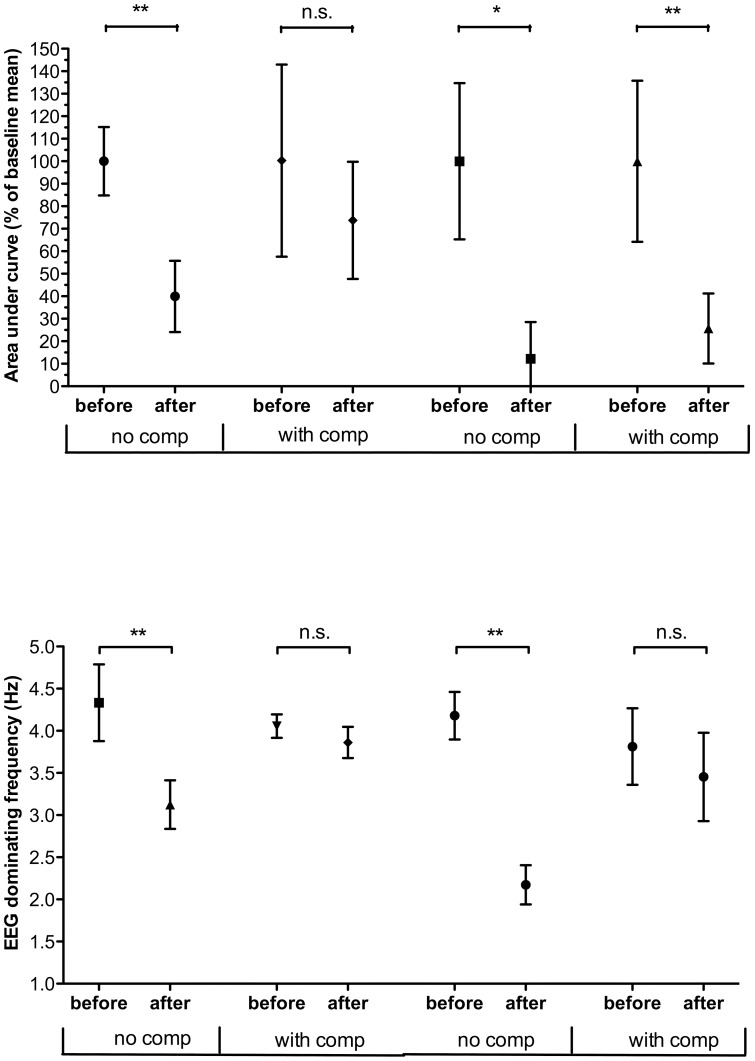
The effect of Morphine on LCEP and EEG. Normalized grand mean and standard deviation of averaged LCEP area under curve and EEG dominant frequency recordings is shown. “With comp” represents experiments with adjustment of volatile anaesthesia to keep a stable dominant EEG frequency after drug. “before” represents baseline measurements before drug and “after” represents LCEPs elicited after drug administration. Left part of the figure shows data from 1 mg/kg and right part shows data from 3 mg/kg Morphine. The results from student's t-test are also shown. See methods section for details.

The effect of Morphine on LCEP and EEG was studied in 22 rats. Systemic administration of 3 mg/kg Morphine (five rats) clearly reduced the averaged LCEP (AUC 12% of control, Student's t-test P = 0.0162, df = 4) as well as reduced the EEG dominant frequency from 4.2 to 2.2 Hz (Figure 4). In contrast to the findings for Midazolam, Morphine 3 mg/kg (five rats) still caused a profound reduction of averaged LCEP (25% of the control AUC, Student's t-test P = 0.0026, df = 4) when the EEG dominant frequency was kept stable. A lower dose of 1 mg/kg Morphine (six rats) also significantly reduced the averaged LCEP (AUC 39% of control, Student's t-test P = 0,0014, df = 5) as well as reduced the EEG dominant frequency (4.3 to 3.1 Hz). However, administration of 1 mg/kg Morphine, when the EEG dominant frequency was kept stable (six rats), did not result in a significant effect on the averaged LCEP as compared to control (AUC 74% of control, P = 0,2514, df = 5).

## Discussion

The present study demonstrates that both EEG dominant frequency and LCEP strongly depends on the level of isoflurane/nitrous oxide anesthesia. Moreover both sedatives (Isoflurane and Midazolam) as well as an analgesic (Morphine, 2 different doses) significantly depress EEG dominant frequency and LCEP. These results indicate that sedation needs to be accounted for when testing the potential analgesic effect on LCEP of a given drug. Importantly, the depressant effect of the sedative Midazolam (10 μmol/kg) on LCEP is abolished if the dominant frequency of EEG is kept stable by adjusting the Isoflurane level, whereas the depressant effect of Morphine 3 mg/kg on LECP was still significant after an analogous adjustment of the Isoflurane level. These findings, as well as the notion that recordings of LCEP may be useful to assess the analgesic properties of a drug provided the dominant frequency of EEG is kept stable, are discussed below.

### Effects of sedation on LCEP and dominant frequency of EEG

In the present study, rats were anaesthetized by Isoflurane and nitrous oxide such that the mean dominant EEG frequency was kept close to 4 Hz and clear LCEPs could be evoked. Increasing the Isoflurane level from 0.9% to 1.1% and 1.3% slowed down the EEG rhythm and, in parallel, depressed the magnitude of the LCEP. Midazolam 10 μmol/kg *i.v*. had similar effects on LCEP and EEG indicating a sedative effect of the compound [31]. Midazolam is a well-known Benzodiazepine that binds to GABA-a receptors and increase the chloride current of the receptor, thus resulting in depression of neural activity [32]. The main effect of benzodiazepines is sedative [32]–[33], but depending on mode of administration have been claimed to show or not to show analgesic or analgesic-like effects [34]. For example, intravenously administered benzodiazepines are not generally considered to have analgesic properties [35], while intrathecal administration, using reflex tests, have been claimed to result in antinociception [36]. The fact that even low doses of benzodiazepines often produce significant sedation has made the interpretation of data more difficult as most studies have used behavioural nociceptive tests, which are easily affected by changes in the state of awareness of the animal. An important finding in the present study was that at the dose tested the depressant effects of Midazolam on both LCEP and EEG dominant frequency was very similar to the effect of adding 0.2% Isoflurane (see Figure 1) and that this effect was reversed by lowering the Isoflurane level by 0.2% (Figure 3). Since there was no differential effect at this dose of Midazolam on LCEP and EEG it appears that the effect of Midazolam at the dose level tested was due to its sedative properties. These findings also support the notion that anaesthetics cause additive effects [37] and consequently that, in an anaesthetized situation when a second sedative is added, the increased sedation caused by the second compound can be reversed by lowering the dose of the first sedative. The present results are pointing towards a possible method to differentiate between sedative and analgesic properties of a drug by compensating for the effects on EEG dominant frequency. It should, however, be kept in mind that two sedatives rarely have identical modes of pharmacological action and thus the issue of interactions between different sedatives is highly complex [38]. Moreover, although the dominant frequency of EEG is a reasonable first choice to measure anaesthesia, it will be important to validate the method using other methods of EEG analysis in subsequent studies.

### Effects of analgesics on LCEP and dominant frequency of EEG

Perhaps the most common analgesic used to attenuate clinical pain in humans and nociceptive behaviour in animals is Morphine [39]–[42]. Morphine is an opioid receptor agonist with high affinity towards the mu-receptor subclass [40]. The analgesic effect of Morphine is thought to origin from both spinal and supraspinal mechanisms. The present study shows that LCEP is depressed by systemic Morphine and thus confirms previous data [15], [18], [43]. However, since Morphine also exhibit sedative properties [44]–[45], the possibility that the entire effect on LCEP is due to its sedative properties could not be excluded. In the present study, the finding that administration of 3 mg/kg, but not 1 mg/kg of Morphine, produced a statistically significant effect on the LCEP also when the dominant EEG frequency was kept stable, indicates that the effect on LCEP of Morphine, at least for the higher dose tested, is partly due to its analgesic properties.

However, the finding that the effect of Morphine (1 mg/kg) on LCEP was significantly reduced when compensating for the effects on the dominant frequency of EEG may suggest that sedation also contributes to the effect of morphine on the LCEP.

## Conclusion and Suggestions

The present study is part of a series of studies that aims at developing a new animal model for studies of pain and analgesia. In screening of potentially useful analgesics, it is of utmost importance to find candidates with as little sedative properties as possible, as most, if not all, available centrally acting analgesics also produce significant sedation. The present findings support the notion that LCEP, provided that changes in EEG is taken into account, could offer a useful tool to assess analgesic properties of systemically administrated drugs. Subsequent studies using different drug regimes will however be needed for validation.
